# ALK inhibition activates LC3B-independent, protective autophagy in EML4-ALK positive lung cancer cells

**DOI:** 10.1038/s41598-021-87966-6

**Published:** 2021-04-27

**Authors:** Anna M. Schläfli, Igor Tokarchuk, Sarah Parejo, Susanne Jutzi, Sabina Berezowska, Nikolai Engedal, Mario P. Tschan

**Affiliations:** 1 Institute of Pathology, University of Bern, Bern, Switzerland; 2 Graduate School for Cellular and Biomedical Sciences, University of Bern, Bern, Switzerland; 3 Department of Laboratory Medicine and Pathology, Institute of Pathology, Lausanne University Hospital and University of Lausanne, Lausanne, Switzerland; 4 Centre for Molecular Medicine Norway (NCMM), University of Oslo, Oslo, Norway; 5 Department of Tumor Biology, Institute for Cancer Research, Oslo University Hospital, Oslo, Norway

**Keywords:** Cancer, Cell biology

## Abstract

ALK inhibitors effectively target EML4-ALK positive non-small cell lung cancer, but their effects are hampered by treatment resistance. In the present study, we asked whether ALK inhibition affects autophagy, and whether this may influence treatment response. Whereas the impact of targeted therapies on autophagic activity previously have been assessed by surrogate marker proteins such as LC3B, we here thoroughly examined effects on functional autophagic activity, i.e. on the sequestration and degradation of autophagic cargo, in addition to autophagic markers. Interestingly, the ALK inhibitor Ceritinib decreased mTOR activity and increased GFP-WIPI1 dot formation in H3122 and H2228 EML4-ALK^+^ lung cancer cells, suggesting autophagy activation. Moreover, an mCherry-EGFP-LC3B based assay indicated elevated LC3B carrier flux upon ALK inhibition. In accordance, autophagic cargo sequestration and long-lived protein degradation significantly increased upon ALK inhibition. Intriguingly, autophagic cargo flux was dependent on VPS34 and ULK1, but not LC3B. Co-treating H3122 cells with Ceritinib and a VPS34 inhibitor or Bafilomycin A1 resulted in reduced cell numbers. Moreover, VPS34 inhibition reduced clonogenic recovery of Ceritinib-treated cells. In summary, our results indicate that ALK inhibition triggers LC3B-independent macroautophagic flux in EML4-ALK^+^ cells to support cancer cell survival and clonogenic growth.

## Introduction

Lung cancer accounts for the largest number of cancer deaths worldwide. It can be divided into small cell and non-small cell lung cancer (NSCLC)^1^. Several driver mutations and oncogenic gene fusions have been identified, such as EGFR, c-MET, ROS1 and KRAS mutations^2^, and rearrangements of ALK, RET, and ROS1^3^, which are in the era of precision medicine inhibited by targeted therapies. In the present study, we focused on lung cancers harboring the EML4-ALK fusion protein, found in 2–9% of all NSCLC patients. The ALK gene encodes a transmembrane tyrosine kinase protein^4^, and the EML4 gene belongs to a large family of proteins, which are associated to microtubules^5^. Different variants of the EML4-ALK gene have been identified, all containing the entire kinase domain of ALK and minimally the N-terminal coiled-coil region of EML4, required for constitutive self-activation of ALK^6^. Downstream of EML4-ALK the PI3K/AKT, JAK/STAT and the MAPK pathway are activated^6^.

In the clinic, ALK fusions are targeted by tyrosine kinase inhibitors. The first generation ALK inhibitor is Crizotinib, which also blocks c-Met^7^. Although primary response rates are promising, resistance to treatment occurs rapidly^4^. In the present study, we used the 2^nd^ generation ALK inhibitor Ceritinib. Ceritinib is more potent than Crizotinib in both in vitro enzymatic assays and in xenograft experiments in mice^8^. Furthermore, Ceritinib is active against some of the common Crizotinib-resistant ALK mutations^8^. In patients, Ceritinib treatment has been shown to prolong progression-free survival compared to Crizotinib^4^. Although Ceritinib is a preferred alternative to Crizotinib, almost all patients develop resistance also to Ceritinib^4^. Another 2^nd^ generation ALK inhibitor, Alectinib, has been approved for first-line treatment of advanced ALK-positive NSCLC due to its superior activity in the CNS and that it may overcome resistance to Crizotinib. However, acquired resistance to Alectinib occurs, and remains problematic. As for Crizotinib and Ceritinib, resistance to Alectinib commonly develops due to ALK mutations, as it was observed in 53% of the tumors from a large cohort of two pooled clinical trials^9^. In the other ~ half of the patients, the resistance mechanisms were not clear. No randomised trials have compared Alectinib with the other 2^nd^- or 3^rd^-generation ALK TKIs. The 3^rd^ generation ALK-inhibitor Lorlatinib is promising for overcoming progression on 2^nd^ generation inhibitors Ceritinib and Alectinib, but not all patients respond^10^. Chemotherapy is still the backbone of NSCLC, including in ALK-positive patients progressing after ALK TKIs without actionable resistance mutations. This clearly warrants evaluation of alternative means to overcome resistance to ALK-inhibition. A cellular process, which may support drug resistance, is autophagy^11, 12^. Autophagy is a cellular degradation and recycling pathway active at basal levels in all cells. Macroautophagy (referred to as autophagy from here on) is the type of autophagy that has been best studied and involves the production of double-membrane vesicles, termed autophagosomes. These specialized vesicles are dedicated to transport cytoplasmic cargos, such as proteins or damaged organelles to lysosomes, where autophagosomes fuse with lysosomes and are degraded together with their load^13^.

Experimental evidence generally supports the notion that autophagy suppresses oncogenic transformation by preserving cellular homeostasis and genomic fidelity^14, 15^. However, in advanced tumors, high autophagic activity may in some cases be proficient for the cancer cells as it can support survival under adverse conditions such as low nutrient and oxygen supply^14, 15^. Cancer therapy adds another complexity, as anti-cancer agents frequently modulate autophagy. Many cancer drugs appear to induce autophagy, that in turn protects the cells^12^. This prompted many researches to combine cancer therapies with autophagy inhibitors to improve therapeutic efficiency^16–19^. On the other hand, autophagy may in some instances be non-protective^20^, or even contribute to drug-induced cell death^21^. Thus, the role of autophagy in cancer cell drug-response seems to be context-dependent. Importantly, however, previous studies on the effects of chemotherapeutic drugs on autophagy are predominantly or exclusively based on analyses of autophagic markers (predominantly LC3) rather than on functional autophagic activity (i.e. the sequestration and degradation of autophagic cargo). Moreover, the role of autophagy has typically been elucidated by interference with autophagy-related (ATG) genes, most of which have non-autophagic roles that may affect cell viability^22, 23^. Thus, the effect of anti-cancer drugs on autophagic activity in cancer cells and its role in treatment responses still remains largely unclear, and requires careful study.

In the present study we aimed at first elucidating if Ceritinib influences autophagy in EML4-ALK positive NSCLC cells, and second whether its modulation affects treatment response to ALK inhibition. We used several methods to determine autophagy and, importantly, autophagic flux, including long-lived protein degradation assay (LLPDA) and lactate dehydrogenase (LDH) sequestration assay. We further assessed the effects of VPS34-inhibition as well as ULK1- and LC3B-knockdown on autophagy. Based on the results from all these assays we conclude that Ceritinib triggers VPS34- and ULK1-dependent autophagy. Interestingly, however, LC3B appeared to be dispensable for Ceritinib-induced autophagy. Lastly, we found that blocking autophagy by VPS34 inhibition significantly decreased cell numbers and clonogenic growth when combined with Ceritinib. In summary our results indicate that Ceritinib induces LC3B-independent, cytoprotective autophagy in EML4-ALK positive NSCLC cells.

## Results

### Ceritinib decreases cell viability and activates autophagy in EML4-ALK positive NSCLC cells

**Figure 1:**
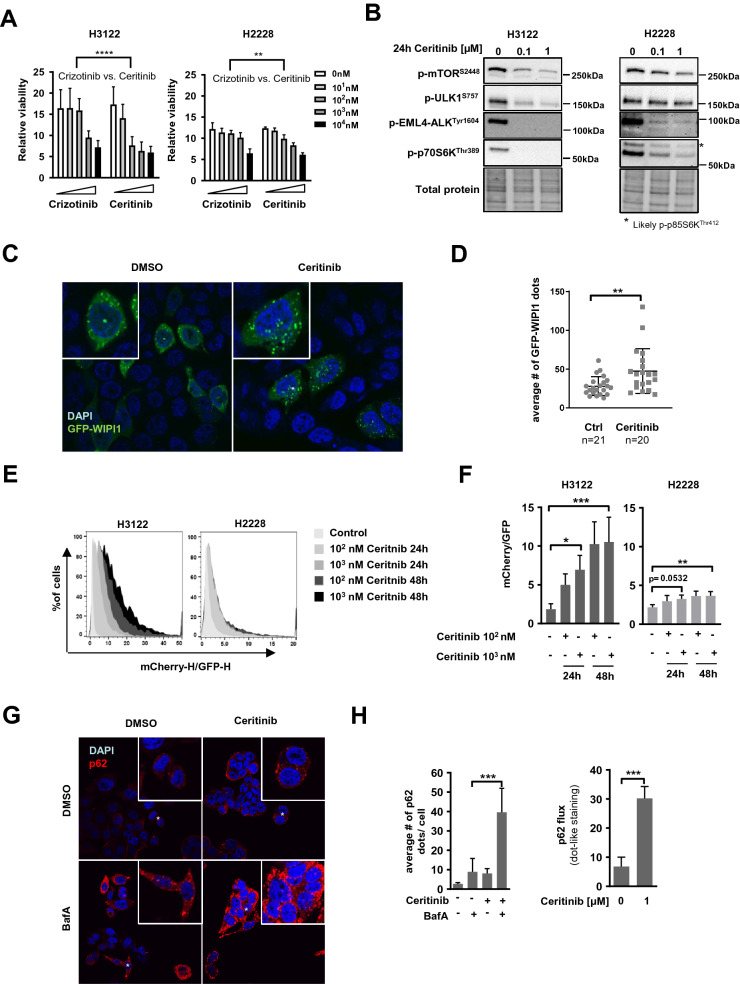
Ceritinib decreases cell viability, abolishes ALK phosphorylation and activates autophagy in EML4-ALK positive NSCLC cells (**A**) H3122 and H2228 cells were treated with Crizotinib or Ceritinib with indicated concentrations for 3 days before metabolic activity was determined using Alamarblue assay (n = 2). Two-Way ANOVA was applied to compare the two drugs. ****p < 0.0001, **p < 0.01. (**B**) Western Blot for p-mTOR, p-ULK1, p-EML4-ALK and p-p70S6K is shown of protein lysate from H3122 and H2228 cells treated with 0, 0.1 or 1 µM Ceritinib for 24 h. Total protein serves as a loading control. (**C**) Confocal microscopy pictures of GFP-WIPI1 transiently over-expressed in H3122 cells treated with DMSO (Ctrl) or Ceritinib for 18 h. (**D**) Quantification of GFP-WIPI1 dots shown in C. Dot plot includes data from 3 independent experiments with 6–7 pictures per experiment (n = 20/21). Mann–Whitney U, **p < 0.01. (**E**) Histograms representing the mCherry to GFP ratio of H3122 and H2228 cells stably expressing the mCherry-EGFP-LC3B construct after Ceritinib treatment (0–1 µM for 24 and 48 h, n = 3). (**F**) Quantification of the ratiometric FACS analysis performed in H. Kruskal–Wallis followed by Dunn`s multiple comparison was applied for statistical testing *p < 0.05, **p < 0.01, ***p < 0.001. (**G**) p62 dots were analyzed by immunofluorescence after H3122 cells were treated with Ceritinib (18 h) ± BafA (last 2 h). Nuclei were counterstained with DAPI. (**H**) Left panel: Bar plot shows quantification of p62 dots shown in G. Experiment has been performed 3 times. Right panel: p62 flux calculated based on the p62 dot quantification by subtracting the BafA untreated sample from its respective BafA treated condition. Mann–Whitney U, ***p < 0.001.

We first aimed at determining the sensitivity of EML4-ALK positive H3122 and H2228 NSCLC cells to ALK inhibition. To this end, we treated the cells with different concentrations of Crizotinib or Ceritinib and performed Alamarblue viability assays. Treatment with increasing concentrations of both drugs for 3 days resulted in a gradual decrease in cell viability (Fig. 1A). In agreement with previous data^18^ we found H3122 (Fig. 1A, left panel) to be more sensitive to ALK inhibition than H2228 cells (Fig. 1A, right panel) and Ceritinib to be more potent than Crizotinib. Our data indicated approximate IC50 values of 1 µM and 10 µM Crizotinib, and 100 nM and 5 µM Ceritinib in H3122 and H2228 cells, respectively (Fig. 1A). H3122 and H2228 cells showed similar sensitivity to growth inhibition upon treatment with another 2^nd^ generation ALKi, Alectinib, whereas H3122 cells were more sensitive than H2228 cells to the 3^rd^ generation ALKi Lorlatinib (Supplementary Fig. 1A). EML4-ALK negative A549 NSCLC cells showed significant resistance to all ALK-inhibitors tested (Supplementary Fig. 1A). Phosphorylation of EML4-ALK at Tyrosine 1604 (p-EML4-ALK^Tyr1604^) was virtually undetectable after 24 h of treatment with 100 nM Ceritinib, Alectinib or Lorlatinib in both H3122 and H2228 cells (Fig. 1B, Supplementary Fig. 1B–D), indicating an efficient and long-term inhibition of ALK. Of note, however, some residual level of p-EML4-ALK^Tyr1604^ could be observed in Ceritinib-treated H2228 cells (Fig. 1B). ALK has been shown to signal via the PI3K/AKT/mTOR pathway in different cell lines^24^. In line with this, Ceritinib induced a slight decrease in mTOR phosphorylation at Serine 2448 and concomitantly a strong reduction in phosphorylation of the downstream mTOR target p70S6 Kinase at Threonine 389 (p-p70S6K^Thr389^) in both cell lines (Fig. 1B, Supplementary Fig. 1B). The levels of the inhibitory phosphorylation of ULK1 at Serine 757 (p-ULK1^S757^, elicited by mTOR) also decreased in Ceritinib-stimulated conditions in H3122 cells (Fig. 1B). Importantly, Ceritinib treatment did not change phosphorylation of mTOR, p70S6 and ULK1 in EML4-ALK negative A549 cells (Supplementary Fig. 1B). Inhibiting EML4-ALK using Alectinib or Lorlatinib resulted in a similar mTOR, p70S6 and ULK1 phosphorylation pattern as seen with Ceritinib treatment (Supplementary Fig. 1C–D).These results suggest that ALK inhibition reduces mTOR activity in H3122 EML4-ALK^+^ NSCLC cells, and that this leads to activation of ULK1 (via removal of the inhibitory phosphate group at Serine 757), which in turn may lead to initiation of macroautophagy. We therefore assessed autophagy activation through analysis of WIPI1 dot formation. WIPI1 is an early autophagy marker, and an increase in WIPI1 dots is associated with autophagy activation^25^. To this end, we transiently over-expressed GFP-WIPI1 in H3122 cells and treated the cells with DMSO or Ceritinib. We found a significantly increased number of WIPI1-positive dots upon ALK inhibition with 1 µM Ceritinib for 18 h (Fig. 1C–D), suggesting autophagy activation. In order to corroborate our data, we further determined autophagy by assessing LC3B carrier flux. LC3B is one of the most commonly used markers to measure autophagy. It is present at autophagic vesicles very early on and stays attached even when the autophagosome reaches the lysosome. To monitor the flux of LC3B to autolysosomal (acidic) vacuoles, tandem fluorescent LC3B constructs such as mCherry-EGFP-LC3B have been developed^26^. Upon arrival to acidic environments (amphisomes and autolysosomes) the EGFP fluorescence is quenched (and degraded), whereas mCherry is more resistant^26^. Using the mCherry-EGFP-LC3B construct, we found a dose- and time-dependent increase in LC3B carrier flux upon Ceritinib treatment in H3122 and H2228 cells (Fig. 1E–F). In agreement with the lower sensitivity of H2228 cells to Ceritinib (Fig. 1A) and the indications of residual p-EML4-ALK^Tyr1604^ levels and mTOR activity in this cell line (Fig. 1B), LC3B carrier flux was less pronounced upon ALK inhibition in H2228 compared to H3122 cells (Fig. 1E–F). Next, we evaluated p62 levels upon ALK inhibition in H3122 cells. p62 is an autophagy receptor that helps in recruiting specific cargo, such as ubiquitinated protein aggregates, to autophagosomes^27^. Upon Ceritinib treatment, p62 dots (visualized by indirect immunofluorescence) increased from about three dots/cell to eight dots/cell (Fig. 1G–H). Importantly, lysosomal blockage using BafA drastically increased the number of endogenous p62 dots/cell, particularly in Ceritinib-treated cells (Fig. 1G–H). Calculating the p62 flux revealed a fivefold higher lysosomal p62 turnover under Ceritinib treatment compared to control conditions (Fig. 1H, right panel).

In summary, our data indicate that Ceritinib is a potent ALK inhibitor, which activates ULK1 kinase via mTOR inhibition. Furthermore, Ceritinib increases autophagy initiation and carrier flux in NSCLC cells, which in light of the involvement of p62 may include selective autophagy.

### Ceritinib triggers ULK1- and VPS34-dependent autophagy

LC3B, p62 and WIPI1 are part of the autophagic machinery, but do not represent autophagic cargo. Moreover, they may be involved in processes other than autophagy. Therefore, these markers alone cannot provide accurate information about autophagic cargo flux.

**Figure 2:**
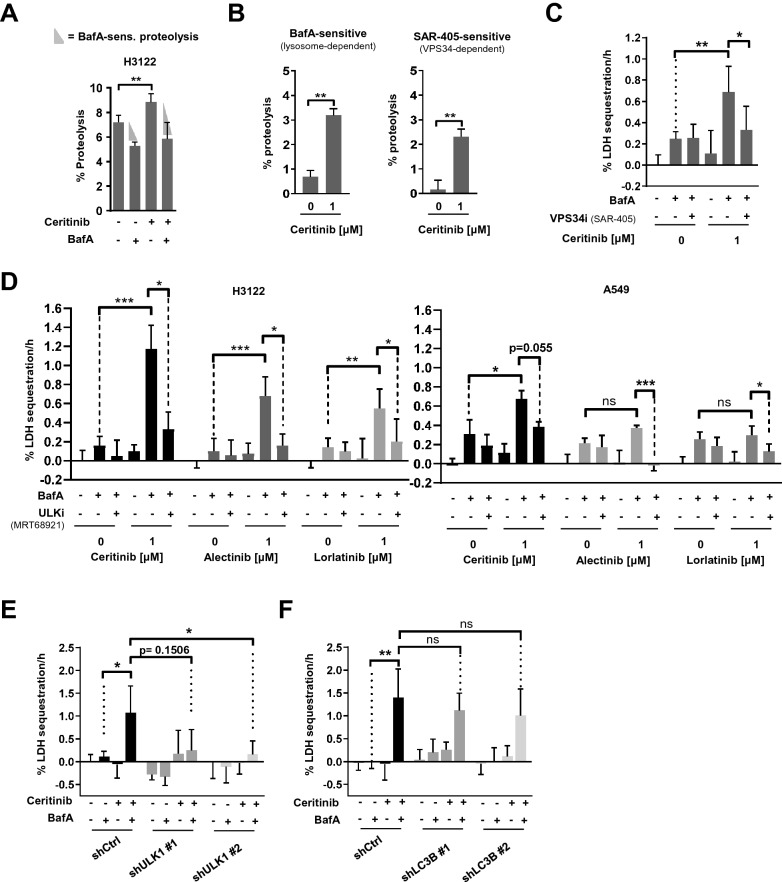
Ceritinib triggers ULK1- and VPS34-dependent autophagy (**A**) Bar plot represents percent proteolysis of H3122 cells after treatment with DMSO or Ceritinib for 24 h in the presence or absence of BafA during the last 5 h (n = 3). (**B**) Quantification of BafA-sensitive and SAR-405-sensitive proteolysis in H3122 cells after treatment with 1 µM Ceritinib for 24 h (n = 3). Percent proteolysis was determined and calculated as described in the methods section. (**C**) H3122 cells were treated with DMSO or 1 µM Ceritinib (24 h) ± BafA and ± SAR405 (during the last 3 h) before LDH sequestration was determined (n = 3). (**D**) H3122 and A549 cells were treated with DMSO or 1 µM Ceritinib/Alectinib/Lorlatinib (24 h) ± BafA and ± MRT68921 (during the last 3 h) before LDH sequestration was determined (n = 3). (**E**) H3122 control (shCtrl) and two ULK1 knock down (shULK1#1 and shULK1#2) cell lines were subjected to LDH sequestration assay after 18 h of Ceritinib treatment. BafA was added during the last 3 h (n = 3). (**F**) Experiment as in E, but with H3122 shCtrl, shLC3B#1 and shLC3B#2 cells (n = 3). Mann–Whitney U was applied to compare two groups; *p < 0.05, **p < 0.01, ***p < 0.001.

Therefore, we next examined autophagy by looking into its function, namely the degradation of proteins. In order to do so, we performed a LLPDA^28^ to compare proteolysis in the lysosomal compartment under basal conditions versus Ceritinib treatment. Ceritinib triggered a significant increase in protein degradation in H3122 cells (Fig. 2A). Importantly, BafA, which blocks lysosome acidification^29, 30^ and thereby function, virtually abolished Ceritinib-induced proteolysis (Fig. 2A). Since lysosome inhibition also affects chaperone-mediated autophagy, we repeated the LLPD assay in H3122 cells using macroautophagy-specific inhibitors, namely SAR-405 or VPS34-IN1, which both target VPS34. Using that experimental set-up, we found that Ceritinib increases both lysosome- and VPS34-sensitive proteolysis, strongly indicating that Ceritinib increases macroautophagic cargo flux (Fig. 2B and Supplementary Fig. 2A–C). In order to substantiate our data, we next assessed autophagy using the LDH sequestration assay. LDH is a cytosolic macroautophagy cargo, and its sequestration by autophagosomes serves as a specific measure of autophagic flux^31^. Autolysosomal inhibitors, like BafA, block LDH degradation, and performing the experimental treatment in the absence or presence of BafA thus allows specific assessment of treatment effects on autophagic sequestration^31^. In line with the LLPDA data, Ceritinib, as well as Alectinib and Lorlatinib, significantly increased LDH sequestration in the presence of BafA, but not in its absence (Fig. 2C–D), indicating that ALK inhibition increases autophagic sequestration activity (autophagosome formation). To strengthen this conclusion, we performed the LDH sequestration assay in the presence or absence of pharmacologic inhibition of macroautophagy using SAR-405 (VPS34 inhibitor). Strikingly, VPS34 inhibition virtually completely abolished Ceritinib-induced LDH sequestration (Fig. 2C), indicating that Ceritinib induces VPS34-dependent autophagy. Next, we used the ULK1/2 inhibitor MRT68921 to validate the VPS34 inhibition data. Similar to VPS34 inhibition, treatment with MRT68921 significantly reduced Ceritinib- as well as Alectinib- and Lorlatinib-induced LDH sequestration (Fig. 2D, left panel). Importantly, control experiments with EML4-ALK-negative A549 cells revealed reduced or no increase in cargo sequestration upon ALK inhibition (Fig. 2D, right panel). Next, we knocked down ULK1 to validate the findings from pharmacologic ULK inhibition. Two H3122 ULK1 knockdown cell lines harboring each a different shRNA to target ULK1 showed strongly reduced LDH sequestration in response to Ceritinib treatment compared to control-transduced cells (Fig. 2E) (albeit the effect of the first shRNA did not reach p < 0.05 statistical significance). Knockdown efficiency for ULK1 was around 80% (Supplementary Fig. 2D). In contrast to ULK1 silencing, knockdown of LC3B failed to significantly reduce Ceritinib-induced LDH sequestration (Fig. 2F), in spite of very efficient depletion (80% and 90% reduction of LC3B-II protein levels with shRNA #1 and #2 respectively (Supplementary Fig. 2E).

Collectively, these data indicate that ALK inhibition induces a canonical form of autophagy (dependent on VPS34 and ULK1) in EML4-ALK positive NSCLC cells, which, however, is independent of LC3B.

### Blocking autophagy via VPS34 inhibition enhances Ceritinib-induced reduction in cell viability and clonogenic growth

**Figure 3:**
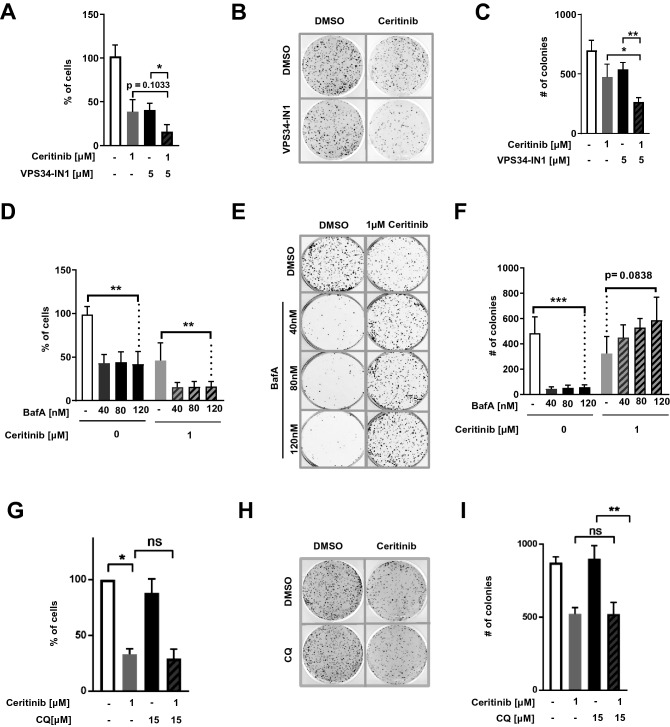
Blocking autophagy via VPS34 inhibition sensitizes cells to Ceritinib (**A**) H3122 cells were treated with DMSO or 1 µM Ceritinib in the presence or absence of VPS34-IN1 for 2 days before living cells were counted and re-seeded for clonogenic assays. Bar plot represents percent of living cells compared to control treated cells (n = 4). (**B**) Clonogenic assay of experiments as described in A. After 2 days of treatment, 5 × 10^3^ living cells were re-seeded and kept for 10 days without treatment. Thereafter, colonies were stained with crystal violet and counted. (**C**) Quantification of the clonogenic assays described in A (n = 4). (**D**) H3122 cells were treated with DMSO or 1 µM Ceritinib in the presence or absence of increasing concentrations of BafA (40, 80 and 120 nM) for 2 days before living cells were counted and quantified from 5 independent experiments as in A. (**E**) Clonogenic assay of cells as described in D. Living cells were re-seeded at low density (5 × 10^3^ cells per well in a 6-well plate) and cultured in the absence of drugs for 10 days. Colonies were counterstained using crystal violet and counted. (**F**) Quantification of colonies shown in E (n = 5). (**G**) H3122 cells were treated with DMSO or 1 µM Ceritinib in the presence or absence of 15 µM chloroquine and asssessed as in A (n = 3). (**H**) Clonogenic assay of chloroquine (CQ) treated cells as described in G and assessed as in B. (**I**) Quantification of colonies shown in H (n = 3).

Having established that ALKi triggers autophagy in EML4-ALK^+^ NSCLC cells, we asked whether its inhibition could increase ALKi-based treatment efficiency. To this end, we challenged the cells with Ceritinib in the presence or absence of the VPS34 inhibitor VPS34-IN1, and determined living cell numbers after 2 days of treatment. The numbers of living cells at day 2 were reduced with the combination as compared to treatment with Ceritinib or VPS34-IN1 alone (Fig. 3A), suggesting that Ceritinib-induced autophagy plays a cytoprotective role in NSCLC cells. Importantly, the combination treatment also significantly reduced the ability of the remaining living cells to form colonies after drug removal (Fig. 3B–C). As a proof of principle, we used BafA to block autophagy at a late stage and repeated the experiment. BafA did indeed mimic VPS34-IN1 in reducing the number of living cells when combined with Ceritinib for 2 days (Fig. 3D), thus supporting the notion that Ceritinib induces protective autophagy in NSCLC cells. Surprisingly, however, the remaining living cells were very potent in recovery from treatment, as shown by the clonogenic assay (Fig. 3E–F). Finally, we used a more clinically relevant late stage autophagy inhibitor, chloroquine (CQ) in combination with Ceritinib. CQ treatment did not significantly reduce H3122 cell viability or clonogenic recovery, either in the absence of presence of Ceritinib (Fig. 3G–I). Together, inhibiting autophagy at an earlier stage with a VPS34 inhibitor clearly enhanced Ceritinib-induced toxicity.

## Discussion

A large number of studies reported that autophagy is induced upon cancer therapy. In this regard, our data are in line with previous findings, including several studies using pharmacological ALK inhibition in different cancer types^32–35^. In all these studies the authors found an increase in autophagy-associated markers, mainly LC3B^32–35^, but the sequestration and degradation of autophagic cargo has not been assessed. Moreover, Torossian A. et al. reported that treating ALK-positive anaplastic large cell lymphoma (ALCL) cells with Crizotinib caused a downregulation of BCL-2 paralleled by increased autophagy. Importantly and different to our study with EML4-ALK positive NSCLC cells, combining Crizotinib with autophagy inhibition to treat ALCL cells did not result in increased cell death, indicating autophagy-associated cell death^36^. Therefore, the type of ALK rearrangement as well as the degree of autophagy activation upon ALK inhibition can determine the type of autophagy modulation required to improve current ALK inhibitor therapies.

Our study is unique in that it thoroughly and conclusively demonstrates that a targeted therapy can enhance full autophagic cargo flux in cancer cells. Moreover, our study is the first to measure autophagy upon Ceritinib-mediated inhibition of EML4-ALK in NSCLC cells. Of note, we also found induction of autophagy, although to a clearly lesser degree, in EML4-ALK negative A549 lung cancer cells treated with Ceritinib but not with Alectinib or Lorlatinib (Fig. 2D). This might be explained by the fact that this drug can also inhibit insulin-like growth factor type 1 receptor (IGF-1R) signalling and thereby attenuate A549 cell growth^37, 38^.

Interestingly, there is a growing list of reports that describe LC3B-independent autophagy^39–43^. To the best of our knowledge, our study is the first to report that a therapeutic anti-cancer drug can induce LC3B-independent autophagy. The ATG8 family, to which LC3B belongs, contains six more members, namely LC3A, LC3B2, LC3C, GABARAP, GABARAPL1 and GABARAPL2^44^. There is a certain degree of specialization among ATG8 family members^44^ but they may also partially compensate each other, and the lack of one member might not be sufficient to block or slow down autophagic flux. In light of this, we would like to emphasize, as it has been done by other groups^40, 45^, that we should not draw conclusions from LC3B-based autophagy assays only.

Rather surprisingly, we found increased autophagic activity using a fluoresecent tagged LC3B assay although Ceritinib-induced autophagy did not require LC3B. We speculate that the autophagosomes that mediate Ceritinib-induced autophagy are LC3B positive and thus the autophagic flux can be measured with an exogenously provided tandem Cherry-EGFP-LC3B construct. LC3B thus seems to be a redundant passenger in Ceritinib-induced autophagy. Most likely, other ATG8 proteins such as the GABARAPs are required instead of LC3B. Similar results have been found in LNCaP cells treated with the ER stressor tunicamycin. Tunicamycin induced LC3B flux as assessed by a tandem fluorescence assay, yet knockdown of LC3B did not reduce autophagic cargo flux^41^. Furthermore, it is possible that LC3B-independent induction of autophagic cargo flux by Ceritinib is cell-line specific. However, LC3B-independent autophagy has been reported in several different cell lines/types, including primary hepatocytes^39^, LNCaP cells^39, 41^ and HeLa cells^42, 43^. In all these cases, autophagy induction was dependent on GABARAP rather than LC3 proteins.

Although, we cannot exclude non-specific drug effects, we believe that the different impact of VPS34-IN1 versus BafA on the colony-formation ability of Ceritinib-treated cells may be related to the fact that VPS34-IN1 inhibits autophagy at an early stage^46^, whereas BafA affects autophagy at a later stage. BafA neutralizes lysosome acidity and thereby function^29, 30^, and eventually reduces the fusion of lysosomes with autophagosomes^29^. 2-days treatment with BafA and Ceritinib will therefore, unlike VPS34-IN1 and Ceritinib, induce a massive accumulation of autophagosomes and inactive autolysosomes in the cells. When the cells are released from the BafA-mediated block upon initiating the clonogenic assay (the drugs are washed out before re-plating), the halted autophagosomes and autolysosomes will be re-activated, and the sequestered cytoplasmic content will be degraded. The extra energy and cellular building blocks that are thus released to the cytosol may very well boost the clonogenic recovery of the cells that had been treated with BafA and Ceritinib. Indeed, this could explain the tendency of BafA to even increase colony formation in Ceritinib-treated cells (Fig. 3F). CQ, on the other hand, was shown to cause a series of cellular alterations in addition to prevent autophagosome-lysosome fusion, possibly explaining its inhibitory activity in colony formation assays compared to BafA^30^.

In terms of improving long-term therapeutic effects of anti-cancer drugs like Ceritinib, VPS34 inhibiton or early-stage autophagy inhibition in general may thus be better suited than blocking autophagy at later stages. In future studies, it will be interesting to explore whether this holds true in general and if applying VPS34 inhibition to the many anti-cancer drugs that have been shown to induce cytoprotective autophagy in various types of cancer cells could improve treatment efficacy^47, 48^. If this is the case, it may help explain the limited clinical success of using the late-stage inhibitor hydroxychloroquine (the only clinically approved autophagy inhibitor so far) in combination with chemotherapy, and point towards novel early-stage autophagy inhibitors, like VPS34-IN1 and others, to hold greater promise for combination therapy with anti-cancer drugs.

## Methods

### Cell lines and culture conditions

H3122 and H2228 cells were obtained from the American Type Culture Collection (ATCC) and were cultured in RPMI-1640 (Sigma-Aldrich, R8785). A549 cells were provided by PD Dr. phil. nat. Thomas M. Marti (Department for BioMedical Research (DBMR), University of Bern, Bern Switzerland) and maintained in Dulbecco’s modified Eagle’s medium/ F-12 Ham (DMEM-F12) (Sigma-Aldrich, D8437). All three cell lines were supplemented with 10% fetal bovine serum (FBS) (Sigma-Aldrich, F7524), 50 U/mL penicillin and 50 µg/mL streptomycin (Sigma-Aldrich, P4333). Futhermore, all cell lines were kept at 37 °C with 5% CO_2_ humidified air and were split 3 times a week using Trypsin–EDTA 0.25% (Sigma-Aldrich, T4049) to detach cells. 293T cells were used for lentivirus production and cultured in DMEM (Sigma-Aldrich, D6046) supplemented with 5% FBS, 1% penicillin/streptomycin, and 1% HEPES (Sigma-Aldrich, H3375) in a humidified incubator with 7.5% CO_2_ at 37 °C.

### Lentiviral vectors

pLKO.1-puro lentiviral vectors expressing shRNA targeting *LC3B (*shLC3B#1 = NM_022818.2-292s1c1, shLC3B#2 = NM_022818.2-247s1c1) or *ULK1* (shULK1#1 = NM_003565.x-535s1c1, shULK1#2 = NM_003565.x-1772s1c1) were purchased from Sigma-Aldrich. Lentivirus production was performed in 293T cells using a 3rd generation packing system described in^49^. The packaging plasmids and the vectors encoding the shRNA of interest were added to 293T cells using the calcium precipitation method. 293T cells were incubated at 5% CO_2_ for 16 h before media was changed. Cells were put back to 7.5% CO_2_ and 48 h later the viral particle-containing supernatants were harvested and passed through a 0.45 µm filter. H3122 cells were infected twice with filtered supernatant. Transduced cells were selected with 1.5 µg/ml puromycin for 3 days, before lowering the concentration to 0.5 μg/ml for another 4 days. After recovery, knockdown efficiency was assessed by western blot analysis and assays of interest were performed.

### Chemicals

Crizotinib (PF-02341066, Selleckchem, S1068), Ceritinib (LDK378, Selleckchem, S7083) and Lorlatinib (PF-6463922, Selleckchem, S7536) were dissolved in DMSO at a stock concentration of 10 mM. Alectinib (CH5424802, Selleckchem, S2762) was dissolved in DMSO at a concentration of 2 mM. The above mentioned chemicals were stored at -80 °C. Bafilomycin A1 (Enzo-Life sciences, BML-CM110) was dissolved in DMSO at a stock concentration of 200 µM. Chloroquine (Sigma-Aldrich, C6628) was reconstituted in distilled water to 10 mM. Both inhibitors were stored at − 20 °C. For autophagic flux measurements, Bafilomycin A1 was used at a final concentration of 200 nM and added during the last 2–5 h of treatment depending on the method used. For the clonogenic assay, chloroquine was added at a concentration of 15 µM and incubated for 48 hr prior to re-seeding. The ULK1 Inhibitor MRT68921 (Sigma-Aldrich, SML1644) was dissolved in water at a final concentration of 20 mM and stored at -80 °C. SAR-405 from ApexBio (A8883) and VPS34-IN1 (S7980) from Selleckchem were dissolved in DMSO to 50 mM and stored at -80 °C. For the LDH experiments, MRT68921 was used at a concentration of 7.5 µM and VPS34-IN1 at 5 µM. Water served as vehicle control for MRT68921, whereas DMSO (Sigma-Aldrich, D4540) was used as a vehicle control for all other drugs dissolved in DMSO.

### Western blotting

Protein lysates were prepared by extracting the total protein from cell pellets using urea lysis buffer (8 M Urea, 0.5% Triton X-100). After protein quantification using Bradford assay (BioRad, 5,000,006) 15–20 µg protein were loaded on 4–20% Mini-PROTEAN TGX Precast Protein Gels (BioRad, 4,561,096) and transferred to PVDF membranes (Trans-Blot Turbo Mini PVDF Transfer Packs, BioRad 1,704,156) using the Trans Blot Turbo Transfer System.

(Bio-Rad Laboratories AG, Pra Rond 23,1785 Cressier). Thereafter, membranes were blocked in either 5% milk or 5% BSA for 1 h at RT while shaking. Subsequently, membranes were put into primary antibody solution: Anti-LC3B antibody (NB600-1384, Novus Biologicals) and p-p70S6K (9234, Cell Signaling) 1:1000 in 5% Milk 3 h RT and p-ALK (Cell Signaling 3341), p-ULK1 (Cell Signaling, 6888), ULK1 (Cell Signaling 4773S) and p-mTOR (Cell Signaling, 5536) 1:1000 in 5% BSA overnight at 4 °C. After washing with TBS-T and TBS, membranes were incubated with HRP-conjugated secondary goat anti-rabbit antibody (Cell signaling) diluted 1:10 000 in 5% milk for 1–3 h at RT. Membranes were washed again and proteins detected using the Clarity Western ECL Substrate (BioRad, 1,705,061) and the ChemiDoc MP system (Bio-Rad Laboratories AG, Cressier) to detect the signal. Saturation of the signal was reported by the software in order to avoid over-exposure of the blots. Quantification of LC3B-II and LC3B carrier flux was performed as described in^44^.

### Microscopy of GFP-WIPI1 dots

For over-expression of GFP-WIPI1, cells were seeded on 18 × 18 mm glass slides (Menzel-Gläser) inserted to 6-well plates (Sarstedt 83.3920.005) at a density of 0.3 × 10^6^ cells/well. 24 h post-seeding, media was changed and GFP-WIPI1 was delivered to cells using Lipofectamine2000 according to the manufacturer’s instructions. We used 4 µg of DNA and 2 µl of Lipofectamine2000 per well. The DNA-Lipofectamine complexes were prepared in a total of 50 µl of Opti-MEM media (ThermoFisher Scientific, 31,985,070). 16 h post-transfection, media was replaced by DMSO- or Ceritinib-containing media. 18 h after treatment, cells were washed, fixed with 4% PFA and mounted as described in^50^. Confocal microscopy pictures were taken with a FluoView-1000 confocal microscope (Olympus Schweiz AG, Volketswil, Switzerland) with a 63 × objective.

### mCherry-EGFP-LC3B ratiometric FACS analysis

The procedure is described in detail elsewhere^50^. Briefly, H2228 and H3122 cells were infected with a lentiviral construct harboring the mCherry-EGFP-LC3B construct. After Puromycin selection, cells were sorted for low to intermediate expression of mCherry fluorescence. For the experiment, 2 × 10^5^ cells were seeded in 6-well plates and were treated with different concentrations of DMSO or Ceritinib the following day. 24 h and 48 h post-addition of Ceritinib, cells were harvested for ratiometric FACS analysis.

### Immunofluorescence staining and confocal microscopy

The principle for p62 immunofluorescence follows the detailed protocol of immunofluorescence staining of LC3B described in^50^. Primary antibody for p62 (Sigma-Aldrich, Clone 2C11, WH0008878M1) was diluted 1:200 in PBS/1% BSA. Confocal microscopy was performed using an Olympus FluoView-1000 confocal microscope (Olympus Schweiz AG, Volketswil, Switzerland) with a 63 × objective. p62 dots, and carrier flux was quantified as described in^44^.

### Long-lived-protein degradation assay (LLPDA)

LLPDA was performed as in^28^, with slight modifications. Cells were seeded in 24-well plates at a density of 2.5 × 10^4^ cells/well. After cells attached, 0.1 μCi ^14^C-valine per ml was added and incubated for 24 h before the addition of Ceritinib. 18 h post-addition of the drug, cells were washed and incubated for 1 h in the presence of 10 mM non-radioactive L-Valine (chasing phase). Thereafter, cells were washed and fresh L-Valine-containing media ± autophagy inhibitors was added. After 5 h, precipitation of proteins in supernatant and cellular fraction was carried out using trichloracetic acid (at final concentration of 10%) and phosphotunctic acid (at final concentration of 1%). After incubation at 4 °C for 30 min, samples were centrifuged at 600 rcf for 15 min to separate the TCA soluble (supernatant) from the TCA insoluble fraction (pellet). Radioactivity in the supernatant and the pellet fractions (after solubilisation in 0.2 M KOH) was determined using a scintillation counter (PerkinElmer). To determine the degradation rate of long-lived proteins the percentage of radioactivity in the TCA-soluble fraction relative to the total radioactivity in both fractions was calculated. BafA- and SAR405-sensitive proteolysis was calculated by subtracting the BafA- or SAR405-containing sample from its corresponding BafA/SAR405 untreated condition.

### Lactate de-hydrogenase (LDH) sequestration assay

A very detailed description of the procedure is described elsewhere^31^. Briefly, H3122 and A549 cells were seeded in a 12-well plate at a density of 2 × 10^5^ or 1 × 10^5^ cells, respectively. After cells attached, either Ceritinib, Alectinib or Lorlatinib was administered at a final concentration of 1 µM for 18 h. DMSO or autophagy inhibitors were added during the last 3 h of incubation. Thereafter, cells were detached, resuspended in 10% sucrose and electrodisrupted using the TX ECM 630 exponential decay wave electroporator (BTX) and the following settings: 800 V, 25 µF, 400 Ω. Cells were immediately transferred to ice-cold after blast buffer (100 mM Sodium monophosphate buffer, 2 mM EDTA, 2 mM DTT, 1.75% Sucrose, pH 7.5). Thereafter the samples were split for determination of total and sedimentable LDH, respectively. Total LDH was extracted by freezing at -80 °C o/n followed by the addition of Triton-X405 (Final concentration 1%). Sedimentable LDH was isolated by spinning the electrodisrupted cell samples, after mixing with 2 × volumes of resuspension buffer (50 mM sodium monophosphate, 1 mM DTT, 1 mM EDTA, and 5.9% sucrose, pH 7.5) supplemented with 0.5% BSA and 0.01% Tween-20, at 18`000 rcf and 4 °C for 45 min. Supernatant was discarded and pellet frozen at -80 °C o/n before the addition of 1% Triton X-405. LDH activity was determined biochemically as the decline in absorbance at 340 nm of nicotinamide adenine dinucleotide (NADH) at 25 °C using a spectrophotometer (TECAN Infinite 200 PRO, Tecan Group Ltd., Männedorf, Switzerland). A standard with defined NADH concentrations was used to calculate the enzyme activity (U/L) by applying the Beer-Lambert law.

### Viability assay

For viability measurements, 2 × 10^3^ cells were seeded in flat-bottom 96-well culture plates. 24 h post-seeding cells were treated with indicated concentrations of different ALK inhibitors, such as Crizotinib, Ceritinib, Alectinib or Lorlatinib. 48 h post-drug-addition metabolic activity was assessed by Alamarblue assay (Thermo Fisher Scientific, DAL1100). Data were obtained by calculating percent reduction of Alamarblue according to the manufacturer’s instructions.

### Trypan Blue exclusion and clonogenic assay

Cells were seeded in 6-well plates at a density of 2 × 10^5^ cells/well. 24 h post-seeding, media was replaced with media containing either DMSO, 1 µM Ceritinib, 5 µM VSP34-IN1, 40, 80 or 120 nM Bafilomycin A1, 15 µM chloroquine, or a combination thereof. 48 h after drug treatment, cells were detached with trypsin–EDTA, resuspended in media, diluted 1:3 with 0.4% Trypan blue, and living cells were counted using a Hemocytometer. For the clonogenic assay, 5 × 10^3^ living cells were re-seeded into 6-well plates and cultured for 10 days in fresh media without drugs. Thereafter, colonies were washed with PBS, fixed with 4% PFA for 2 min and then stained with crystal violet (0.05%) diluted in 30% ethanol for 1 h. After staining, colonies were washed 3 times with water, dried and counted using the GelCount colony counter (Oxford Optronix, Abingdon, United Kingdom).

### Statistical analysis

Graph Pad Prism 7 (GraphPad Software, San Diego, USA) was used to test significance of our data. To compare two groups, the non-parametric Mann–Whitney U test was applied. For comparison of three or more groups Kruskal–Wallis followed by Dunn`s multiple comparison test was applied. Two-way-ANOVA testing was applied to test the difference between Crizotinib and Ceritinib.

## Supplementary Information

Supplementary Information 1.

Supplementary Information 2.
