# Supplementary material for: ALK inhibition activates LC3B-independent, protective autophagy in EML4-ALK positive lung cancer cells
Source: Sci Rep. 2021 Apr 27;11:9011. doi: 10.1038/s41598-021-87966-6 (PMC8079437; doi:10.1038/s41598-021-87966-6)
Supplement: Supplementary file 1 — Supplementary Information 1. [file 41598_2021_87966_MOESM1_ESM.pptx]

## Slide 1
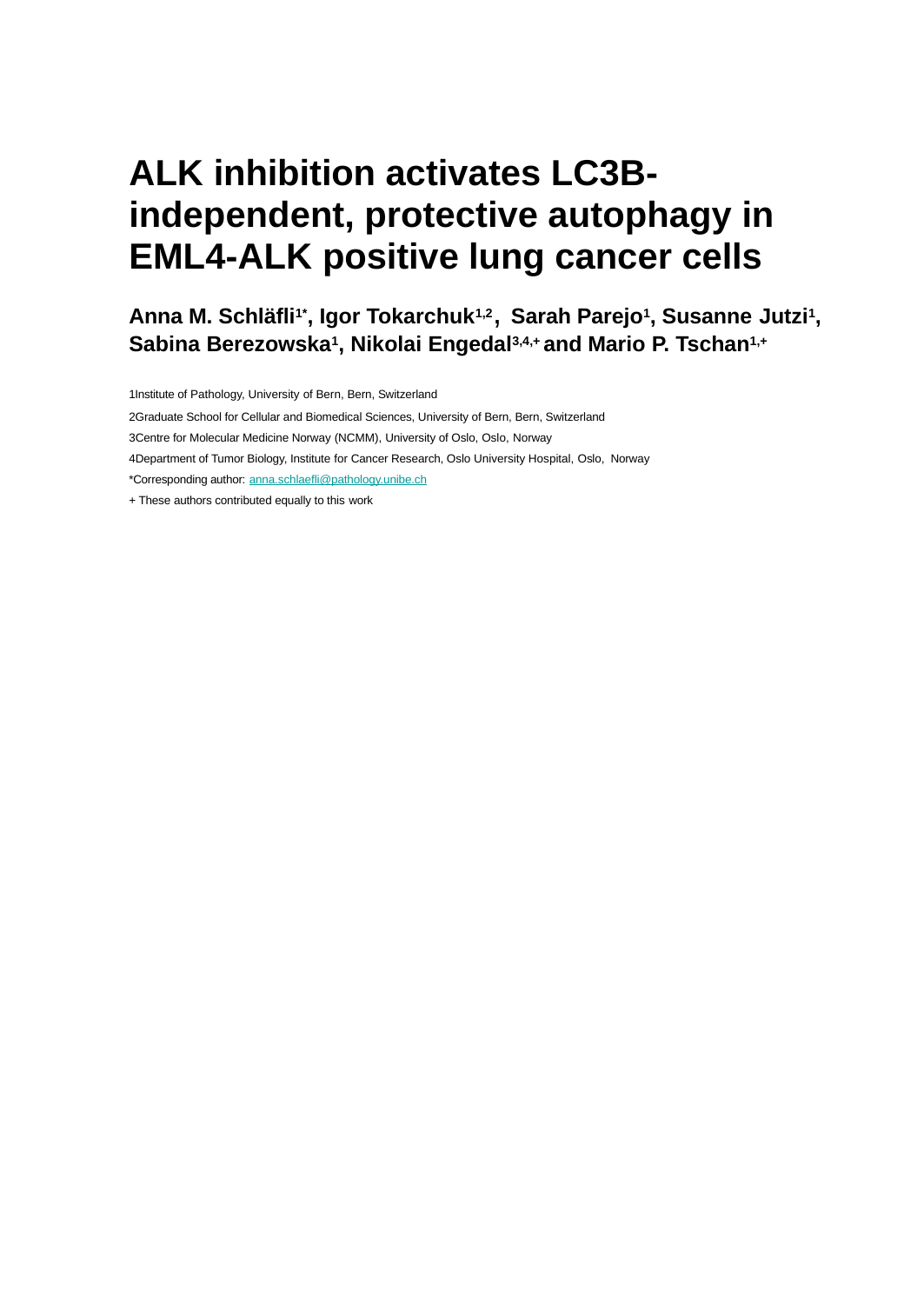

# ALK inhibition activates LC3B- independent, protective autophagy in EML4-ALK positive lung cancer cells
Anna M. Schläfli1*, Igor Tokarchuk1,2, Sarah Parejo1, Susanne Jutzi1, Sabina Berezowska1, Nikolai Engedal3,4,+ and Mario P. Tschan1,+
1Institute of Pathology, University of Bern, Bern, Switzerland
2Graduate School for Cellular and Biomedical Sciences, University of Bern, Bern, Switzerland
3Centre for Molecular Medicine Norway (NCMM), University of Oslo, Oslo, Norway
4Department of Tumor Biology, Institute for Cancer Research, Oslo University Hospital, Oslo, Norway
*Corresponding author: anna.schlaefli@pathology.unibe.ch
+ These authors contributed equally to this work

## Slide 2
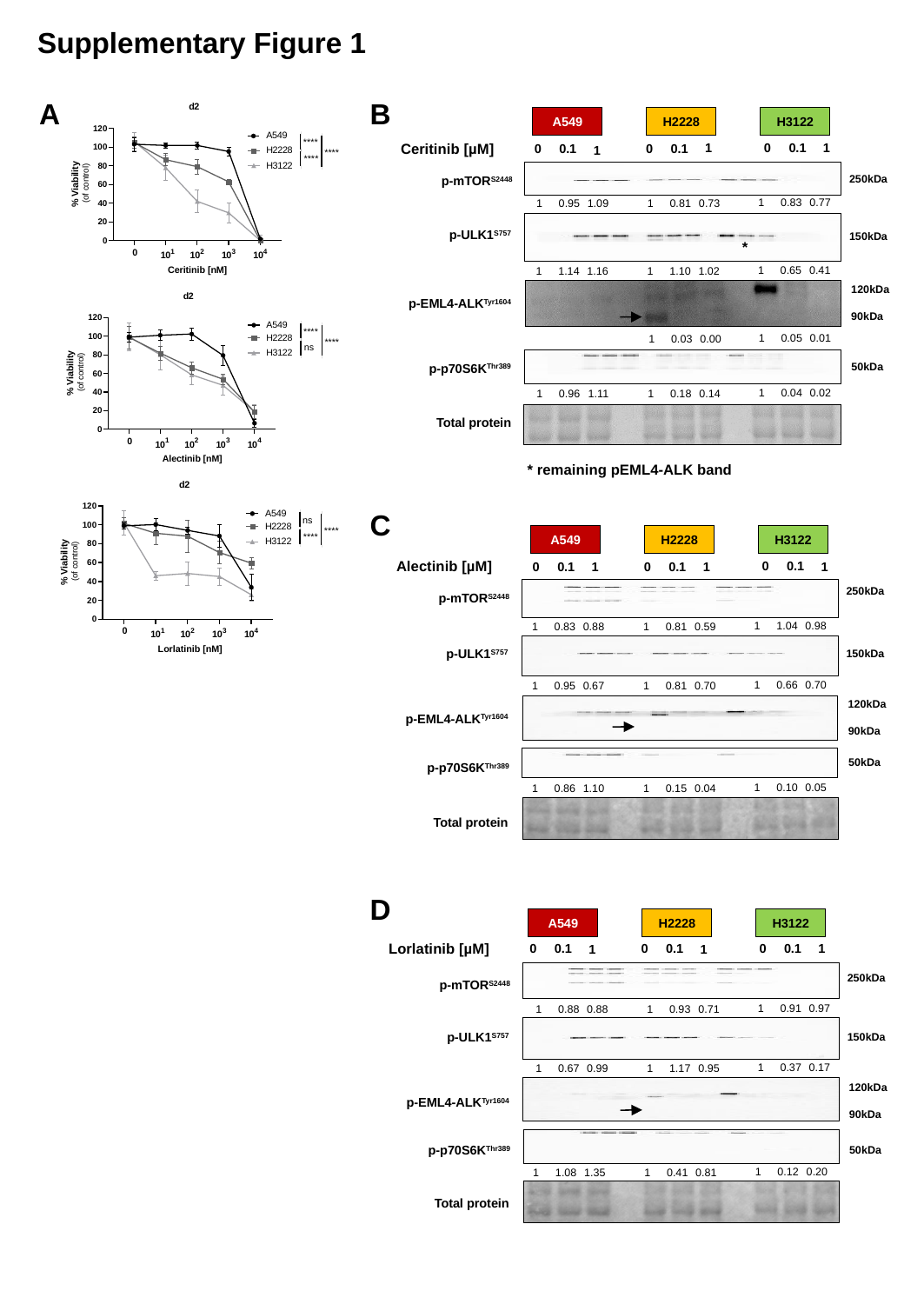

Supplementary Figure 1
A
B
H2228
H3122
A549
Ceritinib [µM]
0.1
0
1
1
0.1
0.1
0
0
1
250kDa
p-mTORS2448
0.83
1
0.77
0.95
0.81
1
1.09
1
0.73
p-ULK1S757
150kDa
*
0.65
1
0.41
1.14
1.10
1
1.16
1
1.02
120kDa
p-EML4-ALKTyr1604
90kDa
0.05
1
0.01
0.03
1
0.00
50kDa
p-p70S6KThr389
0.04
1
0.02
0.96
0.18
1
1.11
1
0.14
Total protein
* remaining pEML4-ALK band
C
H2228
H3122
A549
Alectinib [µM]
0.1
0
0.1
0.1
0
0
1
1
1
250kDa
p-mTORS2448
1.04
1
0.98
0.83
0.81
1
0.88
1
0.59
p-ULK1S757
150kDa
0.66
1
0.70
0.95
0.81
1
0.67
1
0.70
120kDa
p-EML4-ALKTyr1604
90kDa
50kDa
p-p70S6KThr389
0.10
1
0.05
0.86
0.15
1
1.10
1
0.04
Total protein
D
H2228
H3122
A549
Lorlatinib [µM]
0.1
0
0.1
0.1
0
0
1
1
1
250kDa
p-mTORS2448
0.91
1
0.97
0.88
0.93
1
0.88
1
0.71
p-ULK1S757
150kDa
0.37
1
0.17
0.67
1.17
1
0.99
1
0.95
120kDa
p-EML4-ALKTyr1604
90kDa
p-p70S6KThr389
50kDa
0.12
1
0.20
1.08
0.41
1
1.35
1
0.81
Total protein

## Slide 3
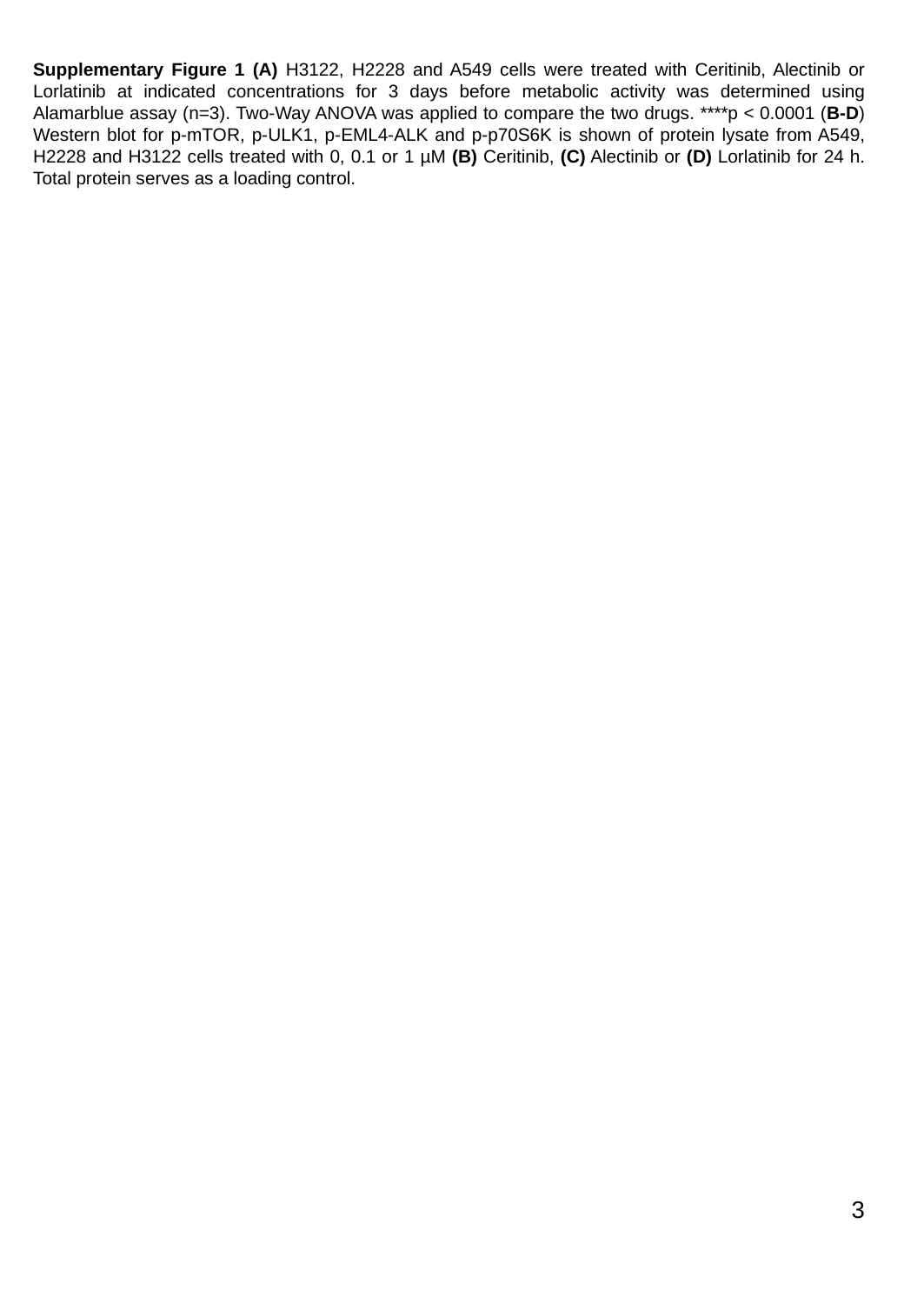

Supplementary Figure 1 (A) H3122, H2228 and A549 cells were treated with Ceritinib, Alectinib or Lorlatinib at indicated concentrations for 3 days before metabolic activity was determined using Alamarblue assay (n=3). Two-Way ANOVA was applied to compare the two drugs. ****p < 0.0001 (B-D) Western blot for p-mTOR, p-ULK1, p-EML4-ALK and p-p70S6K is shown of protein lysate from A549, H2228 and H3122 cells treated with 0, 0.1 or 1 µM (B) Ceritinib, (C) Alectinib or (D) Lorlatinib for 24 h. Total protein serves as a loading control.
3

## Slide 4
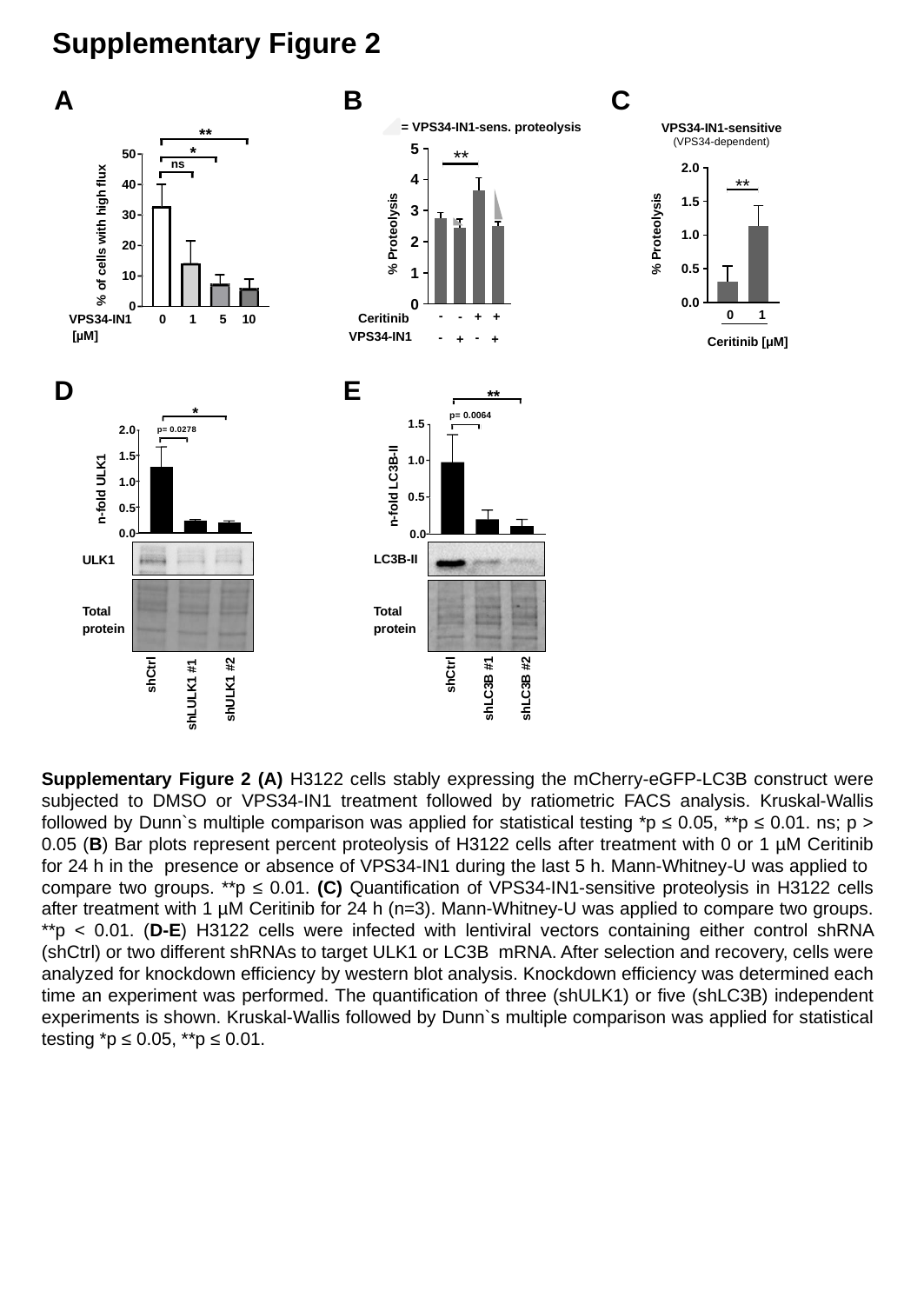

Supplementary Figure 2
B
A
C
= VPS34-IN1-sens. proteolysis
VPS34-IN1-sensitive
(VPS34-dependent)
**
*
50
ns
40
30
20
10
0
0	1	5 10
% of cells with high flux
% Proteolysis
% Proteolysis
0
1
-
-
+
+
-
-
+
+
Ceritinib
VPS34-IN1
 [µM]
VPS34-IN1
Ceritinib [μM]
D
E
**
p= 0.0064
1.5
1.0
0.5
0.0
*
2.0
p= 0.0278
1.5
1.0
0.5
0.0
n-fold LC3B-II
n-fold ULK1
LC3B-II
ULK1
Total
protein
Total
protein
shLC3B #1
shLC3B #2
shLULK1 #1
shULK1 #2
shCtrl
shCtrl
Supplementary Figure 2 (A) H3122 cells stably expressing the mCherry-eGFP-LC3B construct were subjected to DMSO or VPS34-IN1 treatment followed by ratiometric FACS analysis. Kruskal-Wallis followed by Dunn`s multiple comparison was applied for statistical testing *p ≤ 0.05, **p ≤ 0.01. ns; p > 0.05 (B) Bar plots represent percent proteolysis of H3122 cells after treatment with 0 or 1 µM Ceritinib for 24 h in the presence or absence of VPS34-IN1 during the last 5 h. Mann-Whitney-U was applied to compare two groups. **p ≤ 0.01. (C) Quantification of VPS34-IN1-sensitive proteolysis in H3122 cells after treatment with 1 µM Ceritinib for 24 h (n=3). Mann-Whitney-U was applied to compare two groups. **p < 0.01. (D-E) H3122 cells were infected with lentiviral vectors containing either control shRNA (shCtrl) or two different shRNAs to target ULK1 or LC3B mRNA. After selection and recovery, cells were analyzed for knockdown efficiency by western blot analysis. Knockdown efficiency was determined each time an experiment was performed. The quantification of three (shULK1) or five (shLC3B) independent experiments is shown. Kruskal-Wallis followed by Dunn`s multiple comparison was applied for statistical testing *p ≤ 0.05, **p ≤ 0.01.
